# Prostate Cancer Stem Cell-Targeted Efficacy of a New-Generation Taxoid, SBT-1214 and Novel Polyenolic Zinc-Binding Curcuminoid, CMC2.24

**DOI:** 10.1371/journal.pone.0069884

**Published:** 2013-09-24

**Authors:** Galina I. Botchkina, Edison S. Zuniga, Rebecca H. Rowehl, Rosa Park, Rahuldev Bhalla, Agnieszka B. Bialkowska, Francis Johnson, Lorne M. Golub, Yu Zhang, Iwao Ojima, Kenneth R. Shroyer

**Affiliations:** 1 Department of Pathology, Stony Brook University Medical Center, Stony Brook, New York, United States of America; 2 Institute of Chemical Biology & Drug Development, Stony Brook University, Stony Brook, New York, United States of America; 3 Department of Chemistry, Stony Brook University, Stony Brook, New York, United States of America; 4 Department of Urology, Stony Brook University Medical Center, Stony Brook, New York, United States of America; 5 Department of Medicine, Stony Brook University Medical Center, Stony Brook, New York, United States of America; 6 Department of Pharmacological Sciences, Stony Brook University, Stony Brook, New York, United States of America; 7 Chem-Master Int. Inc., Stony Brook University, Stony Brook, New York, United States of America; 8 Department of Oral Biology & Pathology, Stony Brook University, Stony Brook, New York, United States of America; The University of Texas M.D Anderson Cancer Center, United States of America

## Abstract

**Background:**

Prostate cancer is the second leading cause of cancer death among men. Multiple evidence suggests that a population of tumor-initiating, or cancer stem cells (CSCs) is responsible for cancer development and exceptional drug resistance, representing a highly important therapeutic target. The present study evaluated CSC-specific alterations induced by new-generation taxoid SBT-1214 and a novel polyenolic zinc-binding curcuminoid, CMC2.24, in prostate CSCs.

**Principal Findings:**

The CD133^high^/CD44^high^ phenotype was isolated from spontaneously immortalized patient-derived PPT2 cells and highly metastatic PC3MM2 cells. Weekly treatment of the NOD/SCID mice bearing PPT2- and PC3MM3-induced tumors with the SBT-1214 led to dramatic suppression of tumor growth. Four of six PPT2 and 3 of 6 PC3MM2 tumors have shown the absence of viable cells in residual tumors. *In* *vitro*, SBT-1214 (100nM-1µM; for 72 hr) induced about 60% cell death in CD133^high^/CD44^+/high^ cells cultured on collagen I in stem cell medium (in contrast, the same doses of paclitaxel increased proliferation of these cells). The cytotoxic effects were increased when SBT-1214 was combined with the CMC2.24. A stem cell-specific PCR array assay revealed that this drug combination mediated massive inhibition of multiple constitutively up-regulated stem cell-related genes, including key pluripotency transcription factors. Importantly, this drug combination induced expression of p21 and p53, which were absent in CD133^high^/CD44^high^ cells. Viable cells that survived this treatment regimen were no longer able to induce secondary spheroids, exhibited significant morphological abnormalities and died in 2-5 days.

**Conclusions:**

We report here that the SBT-1214 alone, or in combination with CMC2.24, possesses significant activity against prostate CD133^high^/CD44^+/high^ tumor-initiating cells. This drug combination efficiently inhibits expression of the majority of stem cell-related genes and pluripotency transcription factors. In addition, it induces a previously absent expression of p21 and p53 (“gene wake-up”), which can potentially reverse drug resistance by increasing sensitivity to anti-cancer drugs.

## Introduction

Prostate cancer (PrC) is the second leading cause of cancer death among men in Western society [1]. It is initially sensitive to androgen deprivation therapy, but more than 70% of patients will face post-treatment recurrence and transition of the disease to an incurable state [2]. For patients diagnosed with androgen-independent PrC, microtubule stabilizers such as Paclitaxel (Ptx; Taxol) are a first-line treatment strategy that is initially effective. However, approaches to treating chemoresistant PrC are currently lacking [3]. After the initial discovery of cancer stem cells, CSCs [4], it became increasingly evident that tumors are organized hierarchically, containing a relatively minor (but varying) population of tumor-initiating cells and a heterogeneous majority of bulk tumor cells at different stages of maturation. In support of the CSC concept of carcinogenesis, a recent study demonstrated that the expression of several commonly used CSC markers, i.e. CD44, CD166 and ALDH-1, as well as the proportion of cells that express these markers, increases with aging [5]. It is an established phenomenon that aging correlates with a sharp increase in the incidence of prostate and many other cancers [1]. Accumulated knowledge clearly indicates that CSCs are responsible for tumor development, maintenance and resistance to standard treatment modalities. Thus, it has been shown for many cancer types that the tumorigenic cells expressing common CSC markers, in particular CD133 and CD44, are exceptionally resistant to conventional anti-cancer drugs (such as 5-FU, oxaliplatin, irinotecan, docetaxel and others). Moreover, the number of such cells can be significantly propagated after therapy [6-13], which usually manifests as more drug-resistant and more aggressive recurrent and metastatic disease. Therefore, it is conceivable that CSCs represent the most crucial target in the development of a new generation of anti-cancer drugs.

Preclinical evaluation of the candidate anti-cancer agents is traditionally based on the use of monolayer cultures of the total, unselected cancer cells. However, this in vitro model ignores rare yet functionally significant, highly drug-resistant tumor-initiating cells, and thereby has low relevance to the complexity and pathophysiology of *in vivo* tumor tissues. In addition, it was recently demonstrated that the most commonly used established cancer cell lines have no correlation with original clinical samples, which can lead to critical misevaluation of drug efficacy [14]. This can partly explain the high rate of attrition of candidate anti-cancer agents. Thus, only 5% of agents that have anticancer activity in preclinical development are licensed [15]. Therefore, the identification and characterization of patient-derived CSCs, the development of optimal *in vivo* and *in vitro* preclinical models, and CSC-targeted analyses of the drug-induced alterations represent critical steps in the assessment of novel anti-cancer drugs. However, it is notoriously difficult to establish primary cell lines from prostate carcinomas, and this field remains to be one of the most controversial topics in cancer research [16]. First of all, PrC is a malignancy with a high degree of cellular and molecular heterogeneity, therefore, there are objective difficulties in the isolation of pure cell populations from prostate tissues. At the molecular level, there are currently no definitive markers to prove the malignant or nonmalignant nature of prostate cells, and to distinguish between normal and cancer stem cells. Growing evidence also suggests that CSCs might represent a heterogeneous subpopulation of the tumor-initiating cells [17-23]. Nevertheless, a combination of multiple cell surface markers for initial cell sorting followed by thorough functional characterization of the isolated cell phenotypes can lead to the identification of the most functionally significant (i.e. tumor- and metastasis-initiating, or the most drug-resistant) cell populations.

In our previous studies, we have found that a new-generation taxoid, SBT-1214, induces effective long-term suppression of colon tumor xenografts [24] and dramatically down-regulates the expression of multiple stem cell-relevant genes [25]. Searching for safe agents that can potentially decrease systemic toxicity and further improve the CSC-targeted activities of the SBT-1214, we were motivated by numerous anti-cancer features of the natural phytochemical curcumin (diferuloylmethane). In a large number of studies, curcumin has been reported to amplify the cytotoxic effects induced by diverse chemotherapeutic drugs and, importantly, to inhibit clonogenic capacity and induce pro-apoptotic effects on drug-resistant cells expressing stem cell markers [26]. In particular, curcumin significantly decreases the proliferative potential and increases apoptosis of both androgen-dependent and androgen-independent prostate cancer cell lines [27,28]. However, curcumin has low bioactivity and bioavailability, which stimulated us to develop about 30 structural analogues of curcumin (polyenolic zink-binding agents; PEZBINs), including the current lead compound, CMC2.24, which has higher bioactivity, better solubility and no evidence of toxicity even at high doses [29]. The goal of this study was to test the efficacy of the SBT-1214 as a single agent, and its combination with this novel curcuminoid against primary and metastatic prostate tumor-initiating cells.

## Results

### Establishment and characterization of the spontaneously immortalized primary prostate cancer cell line, PPT2

**Figure 1:**
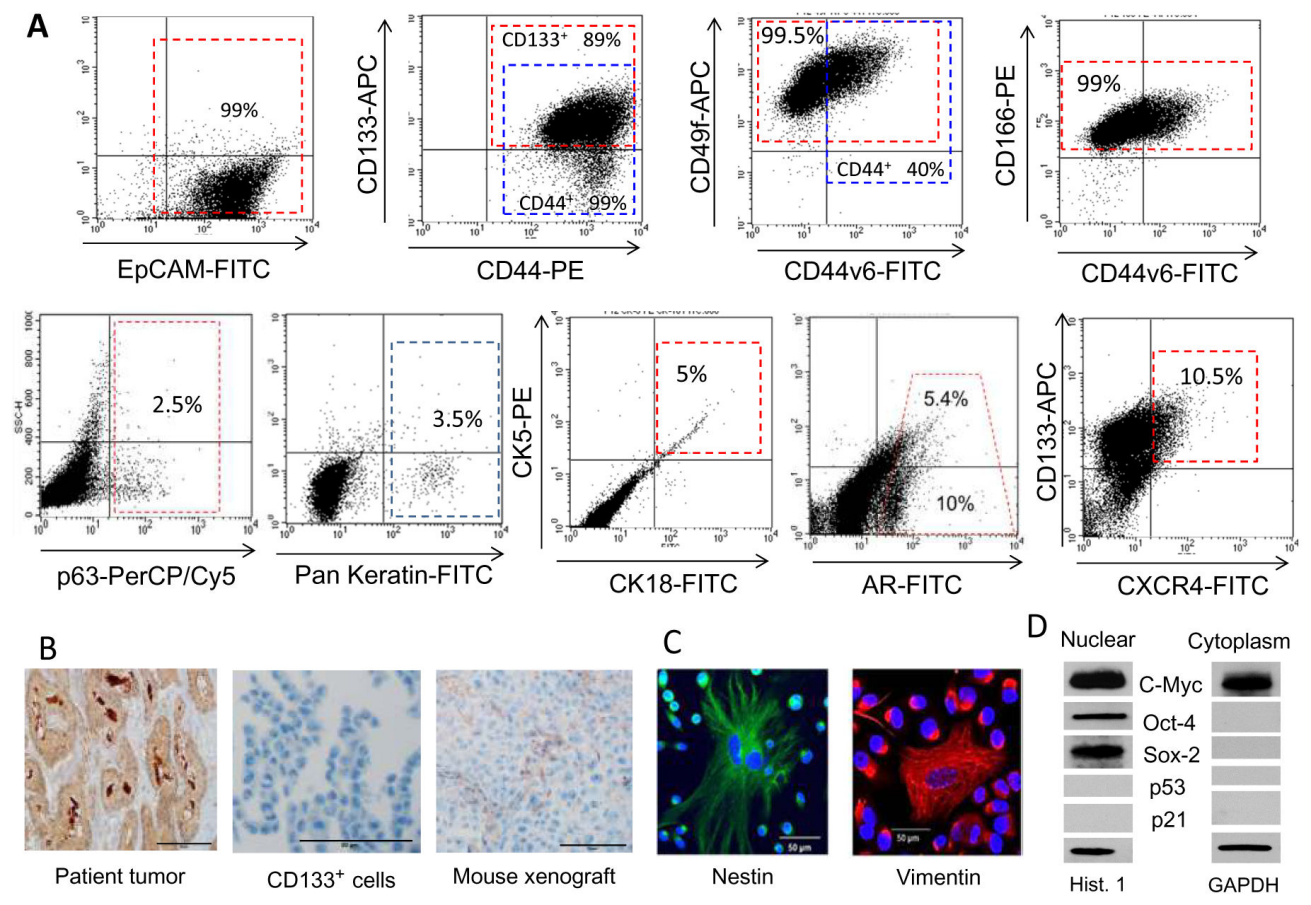
Molecular characterization of the primary prostate PPT2 cell line. (**A**) Representative FACS analyses of the different cell surface markers expression in unsorted PPT2 cells grown for 4 weeks on type I collagen in MSGB medium. Each dotted square represents the population of cells expressing moderate/high levels of a particular marker conjugated with different fluorescent tags. Note a long-term retention of the CD133-APC, standard (CD44-PE) and variant (CD44v6-FITC) CD44 and CD49f-APC. In contrast, only small fractions of the PPT2 cells expressed a marker of basal cells, p63, a marker of differentiated cells, pan-keratin, CK18, AR and CXCR4. (**B**) Immunohistochemical analysis shows that, in contrast to parental tumor tissue, purified CD133^+^ PPT2 cells do not express PSA *in* *vitro*; however, PPT2-induced NOD/SCID tumor xenografts are weakly PSA-positive. (**C**) Immunocytochemical analysis shows uniform expression of vimentin and nestin, with especially high levels of these markers of neural stem cells in large MNCs. (**D**) Western blot analysis shows expression of the pluripotency markers in nuclear and cytoplasmic fractions of the CD133^+^ PPT2 cells. Both the nuclear and cytoplasmic fractions expressed c-Myc and were negative for p53 and p21; only the nuclear fraction expressed Oct-4 and Sox-2.

Dissociated cells of needle biopsies from 22 resected prostate carcinomas of various histological grades were tested for clonogenic and tumorigenic potential *in vivo* and *in vitro* as described in the Methods section. Twenty specimens contained a subpopulation of the fast-adherent cells (FA) to the type I collagen, which initially proliferated in serum-free stem cell medium. Fourteen of the 22 specimens induced floating multicellular aggregates, and only three specimens were able to induce dense 3D spheroids characteristic of CSCs. However, the majority of the primary cells lost their clonogenic and sphere-forming capacities after several passages, which is in line with numerous observations that primary prostate cancer cells have a finite lifespan (5-6 passages) [16]. In contrast, tumor cells isolated from the patient with stage pT2c pNX pMX PrC were spontaneously immortalized and continued long-term *in vitro* and *in vivo* growth (˃28 passages, currently). With serial rounds of transplantation and passaging in a stem cell medium, three different cell populations became evident: the major population of elongated cells, numerous round holoclones containing small cells, and rare, very large multinuclear cells (which we often observe in several established and primary prostate and colon cancer cell lines). Subcloning of these small-cell-containing holoclones led to dramatic enrichment of cells expressing high levels of CD133, CD44, CD44v6, EpCAM, CD49f and CD166 (Figure 1A). This cell line (called PPT2) was serially propagated as NOD/SCID mice tumor xenografts, floating 3D spheroids and type I collagen-adherent cultures. According to the ATCC report (ID number 002872), the PPT2 cells are unique human cells with no match to any profile in the ATCC STR database, which means that they are not contaminated with any known established cell lines. Although phenotype of the CSC-enriched cultures is dynamic due to the dual nature of the CSCs (i.e., ability to self-renew and to generate committed progenitors), the PPT2 cells retain relatively stable phenotypes even up to 8 weeks after the last MACS-CD133^+^ cell sorting and culturing on type I collagen-coated surfaces in serum-free Mesenchymal Stem Cell Growth medium, MSCGM (Lonza; Table 1; Figure 1). Thus, virtually the entire population of PPT2 cells was undifferentiated (only 3-5% expressed marker of differentiated cells, pan-keratin), positive for EpCAM, CD49f, standard isoform of the CD44 (98-99%; clone MEM-85; Invitrogen), and up to 72% express variant isoform, CD44v6 (clone 2F10; R&D). After ˃ 27 passages, about 90% of PPT2 cells still express moderate-to-high levels of CD133 and possess very high sphere-forming capacity in 3D culture containing 1:4 collagen type I/ Stemline Pluripotent Culture Medium (SPCM; Sigma-Aldrich). Twelve-sixteen percent of the PPT2 cells were positive for androgen receptor (AR^+^), and 8-10% were positive for CXCR4 (Figure 1A). In contrast to the original tumor tissue, purified CD133^+^ cells did not express PSA, which appeared again in the NOD/SCID mice tumors after transplantation of the CD133^+^ cells (Figure 1B). The vast majority of the CD133^+^ PPT2 cells expressed high cytoplasmic levels of vimentin and nestin, characteristic of neural and embryonic stem cells. The expression of these two markers was highest in gigantic multinucleated cells (MNCs; Figure 1C). Both nuclear and cytoplasmic fractions of the PPT2 cells expressed c-Myc, whereas other pluripotency markers (Oct-4 and Sox-2) were detected only in nuclear fraction (Figure 1D). Importantly, PPT2 cells were negative for pro-apoptotic/tumor suppressor proteins, p53 and p21, and extremely resistant to standard anti-cancer drugs. By present time, after about 2.5 year of maintenance, the vast majority of the PPT2 cells remains at an immature state, continues to express high ratios and high levels of commonly used stemness and pluripotency markers mentioned above and retains very high tumor-initiating, clonogenic and sphere-forming capacities during serial transplantations of the relatively low cell numbers. All of the above motivated our team to test the two proprietary drugs, SBT-1214 and CMC2.24 against this highly drug resistant, CSC-enriched primary prostate cancer cell line and for comparison, against established a highly metastatic derivative of the PC-3 cell line, PC3MM2 cells, which were characterized in our previous study [30].

**Table 1:** Phenotypic profiling of the PPT2 and PC3MM2 cells with FACS analysis*.

		PPT2 cells**		PC3MM2 cells***	
Marker	Source	Expr. level	% of cells	Expr. level	% of cells
CD133	Miltenyi Bio.	+++	75±15	++/+++	3±1
CD44	Invitrogen MEM-85	+++	99.5±0.5	++/+++	7.5±2.5
CD44v6	R&D Clone 2F10	+++	56±16		
CD49f	BioLegends	+++	99.5±0.5	+++	94±2
CD166	BD Biosci.	+++	99.3±0.5	++/+++	86±8
EpCAM	Miltenyi Bio.	+++	98±0.5	++/+++	83±5
Pan-Kerat	Cell Signal.	++	4±1		
CK5	Santa Cruz	+	7±1	+	3±1
CK18	Santa Cruz	+	5.5±2.5	+	3.5±0.5
CK5/CK18	Santa Cruz	+	4.5±0.5	+	2.5±0.5
p63	Santa Cruz	+	5±2.5	+	7±1
AR	Santa Cruz	+	14±2	++/+++	5±2
CXCR4	R&D	++	10.5±1	+++	12±2

* Mean percentage of cells expressing particular cell surface marker was calculated based on the three independent FACS analyses.

** PPT2 cells (unsorted before analysis, but established by previous repeated MACS-CD133^+^ cell sorting) were cultured on type I collagen-coated dishes in MSCB medium for 2, 4 and 8 weeks.

*** Unsorted PC3MM2 cells were cultured on type I collagen-coated dishes in MSCB medium for 1-2 weeks.

+ ++ and +++ represents low, moderate and high expression.

### Cytotoxic effects of SBT-1214 against CSC-enriched prostate cancer cells *in vitro*

**Figure 2:**
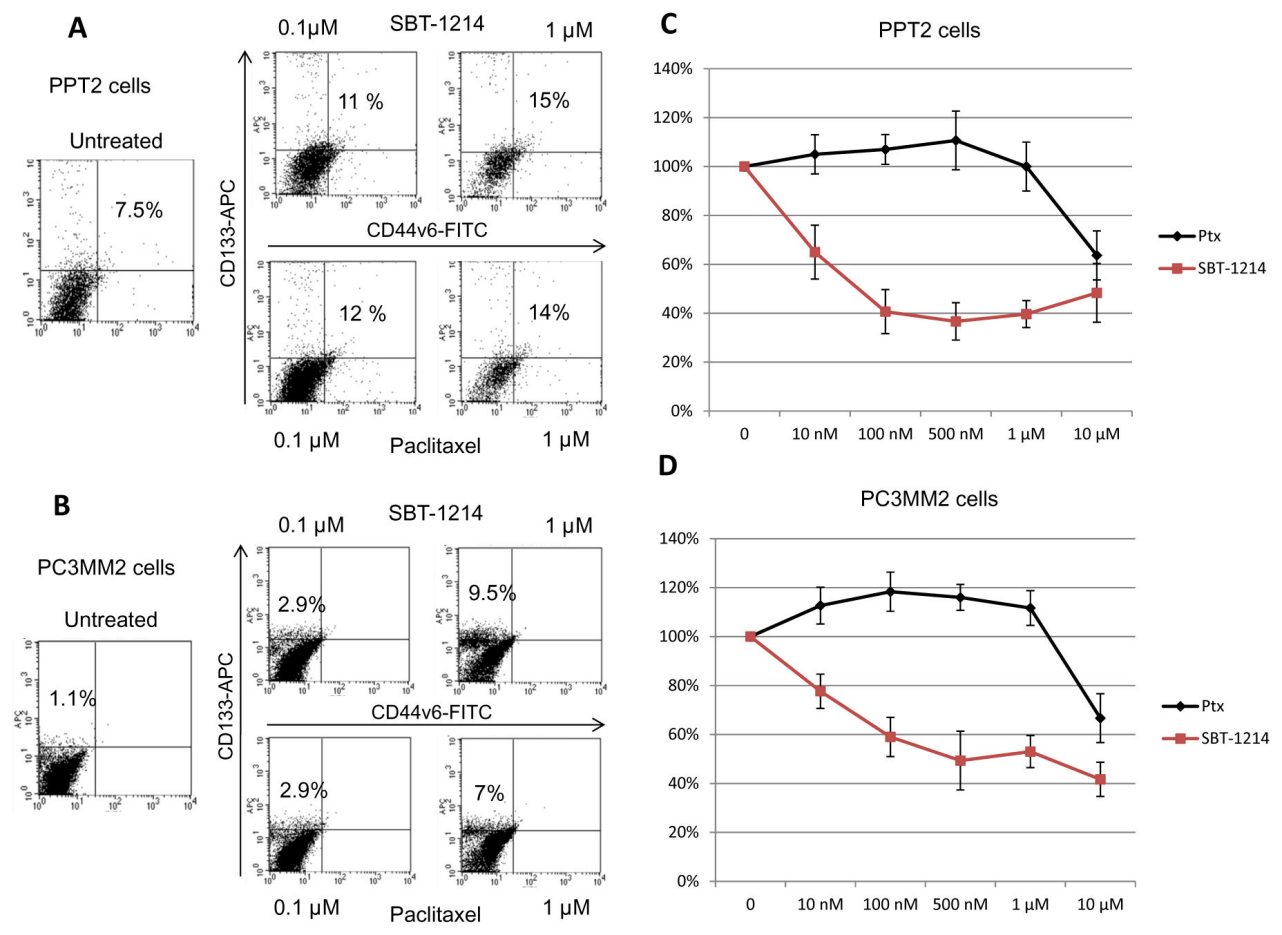
*In vitro* cytotoxicity of SBT-1214 and paclitaxel against prostate CSC-enriched cell populations. Representative FACS analysis shows a dose-dependent increase in the ratios of CD133^high^ PPT2 (**A**) and PC3MM2 (**B**) cells 24 hours after treatment with SBT-1214 and Ptx. Longer treatment (for 72 hours) with SBT-1214 (10 nM-10 µM) induced up to 65% cell death in PPT2 cells (**C**) and up to 60% in PC3MM2 cells (**D**; lower line). In contrast, treatment with the same doses of Ptx did not suppress proliferation of the tumorigenic prostate cancer cells (**C, D**; upper line). Cells were incubated with indicated drug concentrations, and data was obtained with standard MMT assay based on three independent experiments with four repeats in each treatment group. Values are the means±SD.

**Figure 3:**
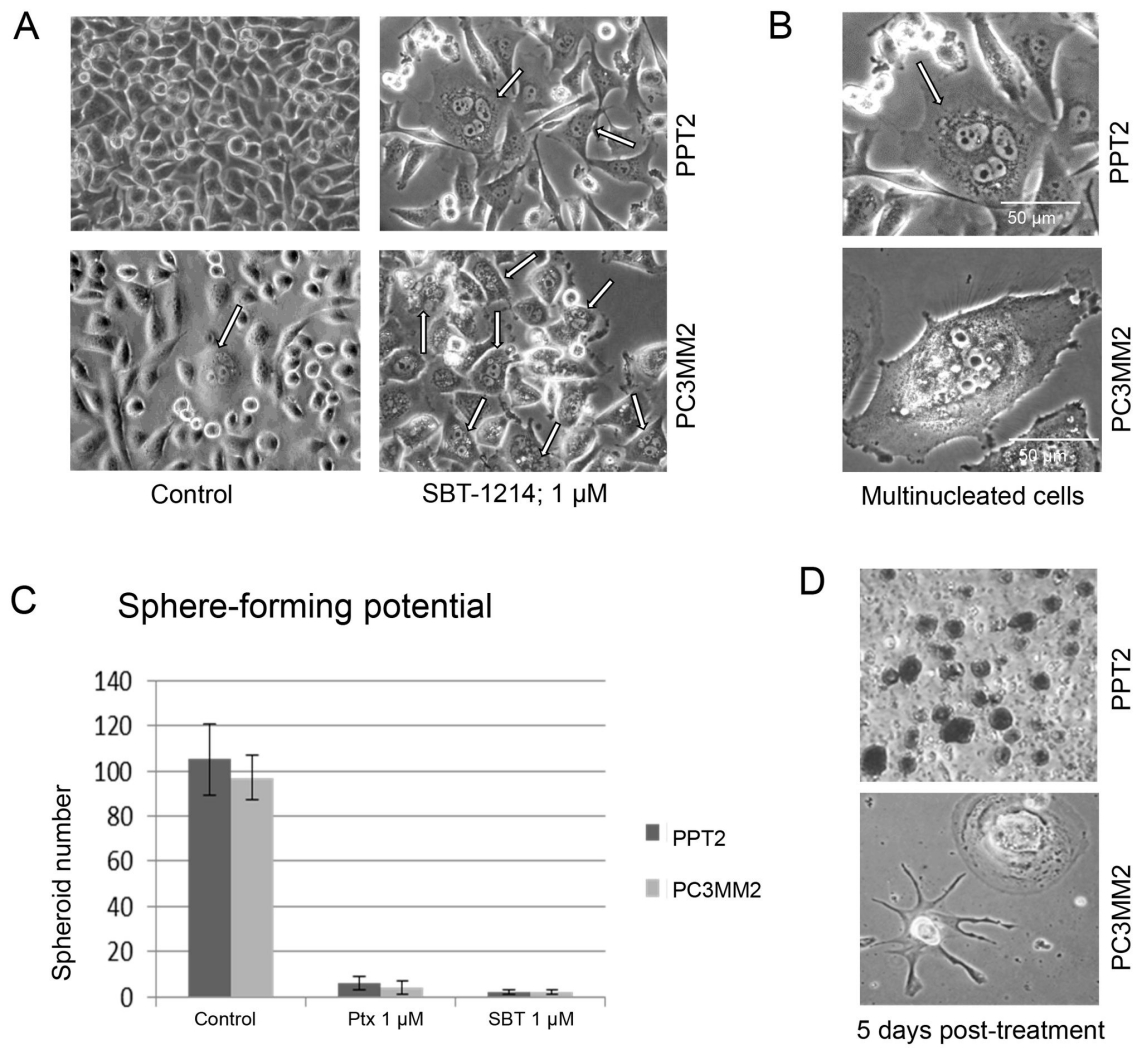
Treatment with SBT-1214 visualized the presence of large multinucleated cells, abolished the sphere-forming capacity of survivor-cells and induced delayed cell death in CD133^+^ prostate cancer cells. (**A**, **B**) Phase contrast microphotographs show that a single treatment with 1 µM SBT-1214 for 48 hr induced death of the PPT2 and PC3MM2 cells with enrichment of the large multinucleated cells (arrows). (**C**) Post-treatment loss of the sphere-forming capacity of survivor-cells compared to untreated ones. Values are the means ±SD based on 3 independent repeats. (**D**) Profound death of treated PPT2 and PC3MM2 cells during the next several days in culture.

Treatment with SBT-1214 and, for comparison, with the commonly used microtubule stabilizing agent paclitaxel (Ptx; Taxol) increased the ratios of cells with highest expression of CD133 in a concentration-dependent manner (from 10nM to 10µM; for 24 hours) in both PPT2 and PC3MM2 cell lines (representative FACS is shown on Figure 2A, B). This data suggests that both drugs preferentially affected cells with low CD133 expression, and initially did not affect or even stimulate proliferation of the CD133^high^ cells. However, a longer treatment (for 48-72 hours) revealed a profound difference between the cytotoxic effects of SBT-1214 and Ptx: while Ptx-treated cells retained viability and the number of viable cells was still increased, up to 60% of the SBT-1214-treated CD133^+^ cells were killed with the same concentrations of the drug (the MTT assay data are shown on Figure 2C, D; Figure 3A). Surprisingly, lower concentrations of SBT-1214 (100nM-1μM) induced higher cytotoxicity in PPT2 cells compared to 10µM drug concentration. Paclitaxel at 10 µM was cytotoxic and induced about 40% cell death (Figure 2C, D). At this time point, an increased ratio of very large MNCs (often ≥200 µm) was obvious in both PPT2 and PC3MM2 cells (Figure 3A, B). To test whether or not treatment with SBT-1214 affects the clonogenic potential of the CD133^+^ cells, PPT2 and PC3MM2 floating spheroids were treated with 1µM of the drug for 24 hours in order to induce alterations, but avoid profound cell death. We have found that in contrast to control untreated spheroids, SBT-1214 treated cells lost their ability to induce both secondary 3D spheroids (Figure 3C) or collagen-adherent colonies (not shown). Both PPT2 and PC3MM2 cells-treatment-survivors exerted profound cell death at 2-5 days post-treatment with SBT-1214 (Figure 3D).

### *In vivo* efficacy of SBT-1214 against prostate tumor xenografts induced by CD133^+^ cell populations

**Figure 4:**
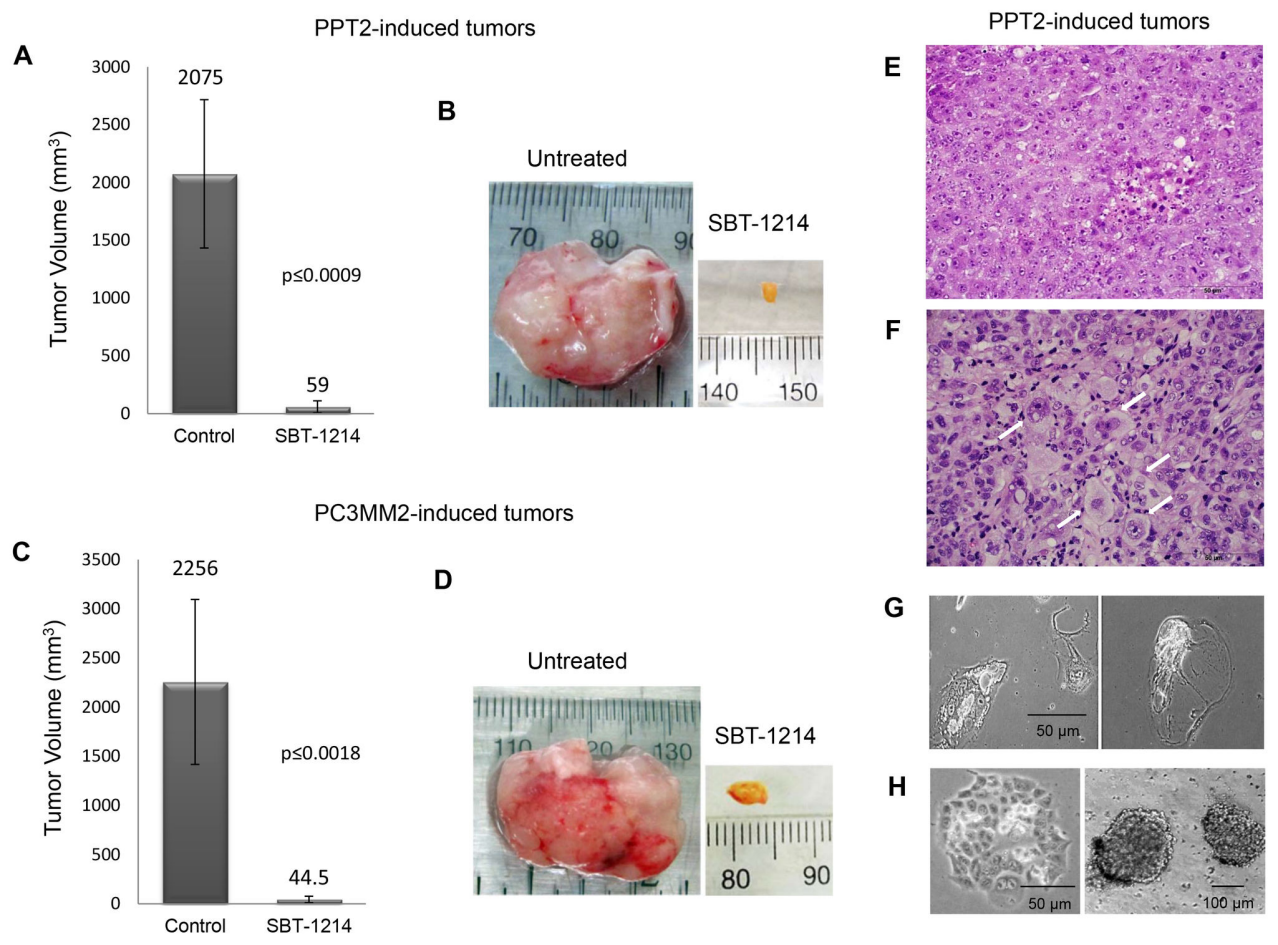
Anti-tumor effects of SBT-1214 *in* *vivo*. NOD/SCID mice were ectopically implanted with 3,000 of CD133^+^ PPT2 and PC3MM2 cells on the flanks. Three weeks after injection, mice were treated with weekly i.v. injections of SBT-1214 (x4: 40, 20, 20, 20 mg/kg). This treatment modality induced dramatic reduction in tumor size in the majority of the PPT2- and PC3MM2-induced tumors (**A**-**D**). Representative cases are shown on B and D. Values are the means ±SD; *p*≤0.0009 for PPT2-induced tumors (SBT-treated versus untreated controls; n=6), and *p*≤0.0018 for PC3MM2-induced tumors (n=6). Histopathological analysis of the residual tumors showed the presence of viable cells and accumulation of large multinucleated cells in 2 of 6 PPT2 tumors and 3 of 6 PC3MM2 tumors (representative H&E staining of untreated and SBT-1214-treated PPT2 tumor tissues is shown on **E, F**). *Ex* *vivo* death of the drug-treated cells in culture (**G**). Control untreated tumor cells retained profound clonogenic and sphere-forming capacities during serial passaging (**H**).

After transplantation of the CD133^+^ PPT2 and PC3MM2 cells, NOD/SCID mice were randomly divided into 3 groups for each cell type: one as an untreated control (n=4), a second group for SBT-1214 treatment with 40, 40, 40 mg/kg weekly regimen (n=4), and a third group for treatment with 40, 20, 20, 20 mg/kg weekly regimen (n=6). Treatment was started 2-3 weeks after transplantation of the tumor cells, when tumor xenografts reached ˃100 mm^3^; tumor development was monitored weekly. Although all four tumors treated with 40, 40, 40 mg/kg weekly regimen had dramatically shrunk by the third treatment, all mice expressed common signs of systemic toxicity and were euthanized (data are not shown). The treatment with 40, 20, 20, 20 mg/kg weekly regimen induced more significant tumor regression (Figure 4A-D), with much lower systemic toxicity. One week after the last treatment, all residual tumors were harvested and analyzed histopathologically and for *ex vivo* clonogenic capacity. Untreated control tumors were removed upon reaching ~2 cm in largest diameter in accordance with the IRB requirements.

#### Histopathological analysis

The hematoxylin and eosin stained tissue sections of the control untreated patient-derived CD133^+^ cell-induced NOD/SCID mice tumor xenografts showed highly atypical epithelial cells forming nests and gland-like structures, consistent with poorly differentiated adenocarcinoma (Figure 4E). Numerous atypical mitotic figures and central necrosis were usually present. The two larger PPT2-induced SBT-1214-treated residual tumors were also diagnostic of poorly differentiated adenocarcinoma, but possessed a greater degree of nuclear atypia, and only few mitotic figures. In addition, the SB-1214-treated tumor showed focal hyalinization but no evidence of necrosis. Smaller residual tumors (n=4) showed a higher level of hyalinization and a lack of viable cells.

#### Post-treatment alterations in clonogenic and sphere-forming capacities

Total cell suspensions from the control and SBT-1214-treated residual tumors were seeded on type I collagen-coated dishes and ULA plates to test for the presence of CSCs and their ability to induce secondary spheroids or adherent cell colonies. Four of six PPT2-induced and three of six PC3MM2-induced SBT-1214 treated residual tumors were composed of tan or dark red masses which did not show the presence of viable cells, and did not produce either adherent or floating colonies in culture. Two of the PPT2-induced post-treated residual tumors and three of the PC3MM2-induced tumors contained viable slowly proliferating cells. The two PPT2 residual tumors also showed clusters of gigantic multinucleated cells-treatment-survivors (arrows on Figure 4F) similar to those commonly observed after treatment of the CSC-enriched populations with chemotherapeutic agents *in vitro* (Figure 3B). However, these cells did not induce any compact spheroids in non-adherent culture conditions, producing only loose multicellular aggregates, which underwent profound cell death in several days of *ex vivo* culturing (Figure 4G). In contrast, dissociated cells from untreated tumor xenografts were serially passaged *in vivo* and *in vitro* (Figure 4H shows type I collagen-adherent holoclone and floating spheroids induced during serial passaging of the untreated PPT2 mice tumor xenograft cells).

### Combination of SBT-1214 with CMC2.24 increases its CSC-targeted cytotoxic and stem cell-modulating activities

**Figure 5:**
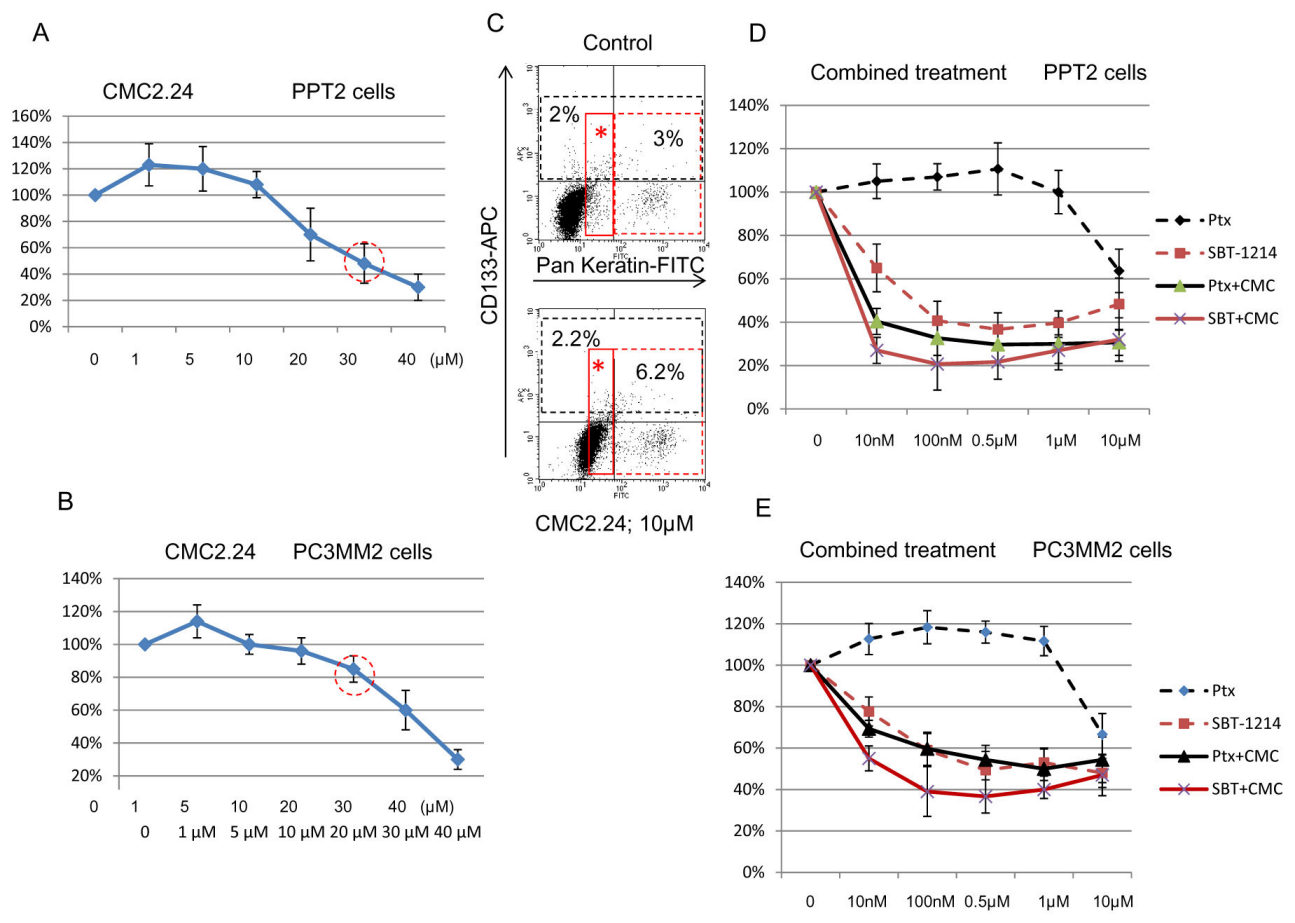
Cytotoxic effects of the SBT-1214/CMC2.24 combination against prostate CD133^+^ cells. (**A**, **B**) The CMC2.24 as a single agent induced bi-phasic effects on prostate cancer cell proliferation: lower concentrations of it promoted proliferation, whereas higher ones were cytotoxic. (**C**) In contrast to SBT-1214, treatment with CMC2.24 did not induce an increase in the ratio of CD133^+^ cells (black dotted areas), but similarly to SBT-1214, increased expression of the differentiation marker pan-keratin (red dotted areas) and shifted the entire cell population toward differentiation (areas with asterisks). A combination of the two agents induced more significant cell death of the CD133^+^ PPT2 (**D**) and PC3MM2 (**E**) cells compared to each compound as a single agent. Data were obtained with standard MMT assay (**A**, **B**, **D**, **E**). Values are the means ±SD of the three independent experiments with 4 repeats for each drug concentration.

To study the potential interactions between SBT-1214 and Ptx with a novel synthetic curcuminoid, CMC2.24, the effectiveness of various drug concentrations and their combination with CMC2.24 was evaluated in the tumor-initiating fractions of the PPT2 and PC3MM2 cell lines. Cell viability was determined by the MTT assay and FACS analysis. We have found that the CMC2.24 (as well as curcumin; not shown) often exerted biphasic effects on prostate CD133^+^ cells. Thus, treatment with low concentrations of CMC (up to 10µM) for 72 hr stimulated cell proliferation, whereas higher doses (10-40µM) were increasingly cytotoxic (Figure 5A, B). FACS analysis revealed that in contrast to SBT-1214 and Ptx, CMC2.24 did not induce an increase in the ratios of cells with highest expression of CD133 (Figure 5C; black dotted area), but led to a significant shift of the entire cell population toward differentiation (C; area with red asterisk) and some increase in cells with highest expression of pan-keratin (C; red dotted area). These data indicate that low concentrations of CMC2.24 increase proliferation of the non-stem progenitors rather than CSCs. Importantly, the combination of 30µM CMC2.24 (which is not toxic even at much higher doses [29]) with low concentrations of SBT-1214 or Ptx (10nM-1µM) exerted more profound death of the CD133^+^ cells than each compound as a single agent (Figure 5D, E). After temporary post-treatment accumulation of the large multinucleated cells, they lost the ability to induce floating 3D spheroids and underwent profound cell death similarly to the SBT-1214-treated cells shown on Figure 3.

### SBT-1214-induced alterations in the stemness-related gene expression profile

**Figure 6:**
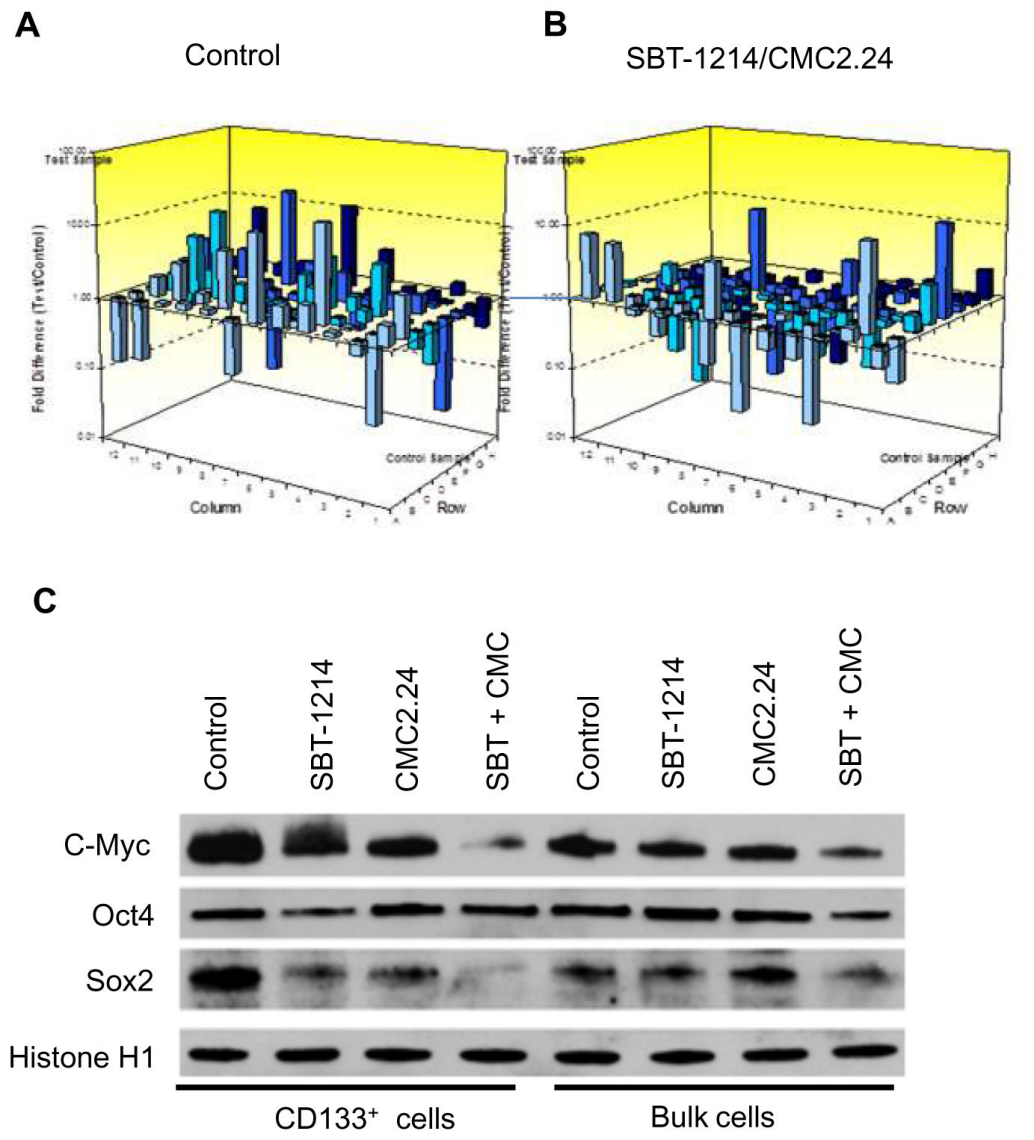
The SBT-1214/CMC2.24 combination induced suppression of the stem cell-relevant transcription factors in CD133^+^ PPT2 cells. (**A**) Multiple stem cell-relevant transcription factors are up-regulated in the CD133+ cell population compared to their differentiated counterparts. (**B**) Down-regulation of the up-regulated transcription factors after treatment with 1 µM SBT-1214 and 10 µM CMC2.24 for 24 hr (PCR array assay; SABiosciences; PAHS 501). Western blot analysis confirmed significant down-regulation of key pluripotency transcription factors, c-Myc and Sox-2 in CD133^+^ cells (**C**). Histon H1 was used as a loading control.

**Figure 7:**
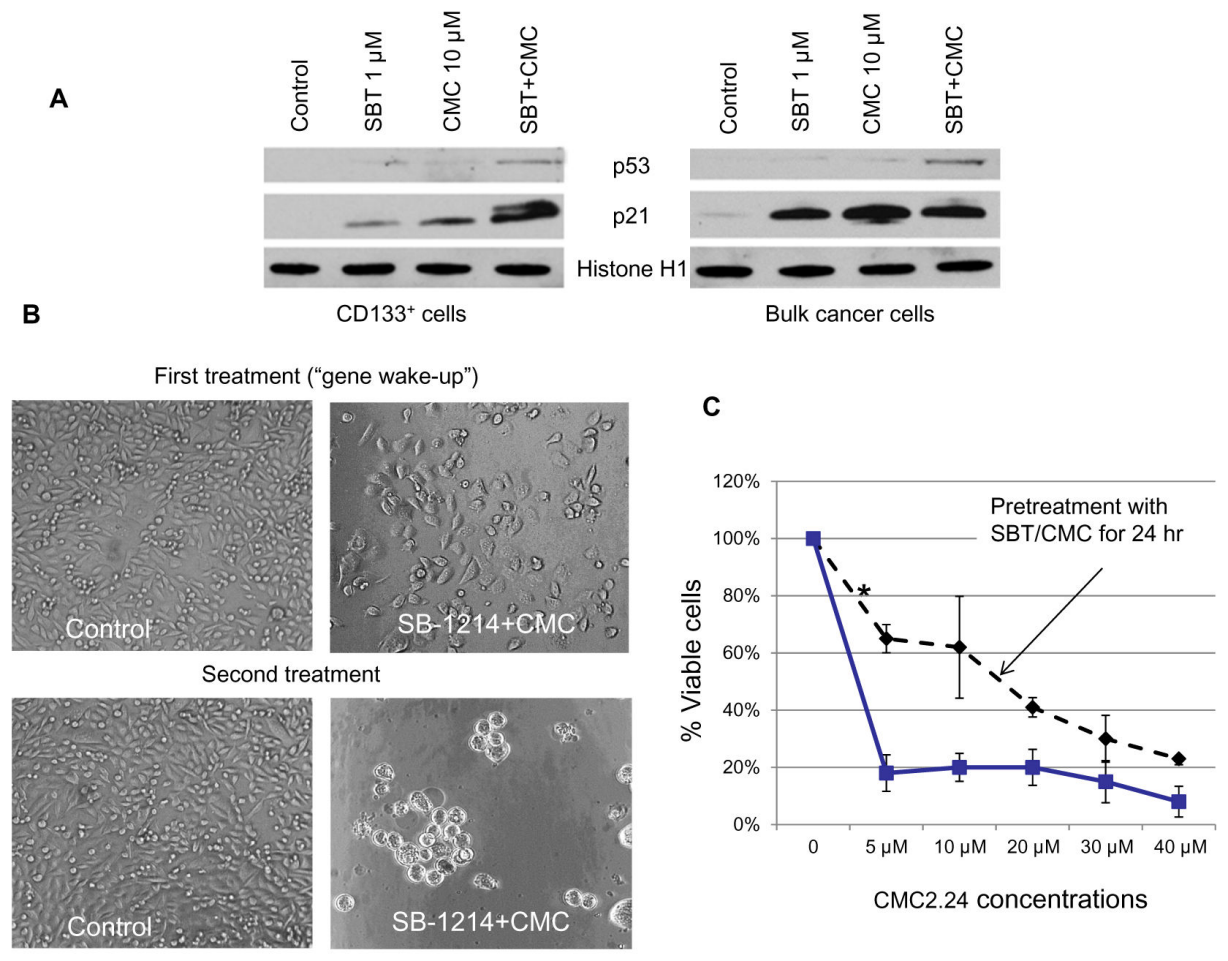
The p53/p21 “gene-wake-up” induced by SBT-1214/CMC2.24 combination. (**A**) Previously absent pro-apoptotic/tumor suppressor proteins, p21 and p53 were induced by single treatment with SBT-1214/CMC2.24 combination for 24 hr in both CD133^+^ and bulk PPT2 cells. Such “gene wake-up” led to significant increase in the sensitivity of the CD133^+^ cells to this drug combination (B, C). Pre-treatment [SBT-1214 (1 µM) + CMC2.24 (10µM)]; second treatment [SBT-1214 (1 µM) + CMC2.24 variable]. Data were obtained with standard MMT assay.

In our previous studies, using genome-wide and pathway-specific gene expression profiling, we have found that the tumorigenic cell populations isolated from the established prostate and colon cancer cell lines expressed upregulated levels of the anti-apoptotic genes, ABC transporters and the majority of stem cell-related genes [25,30,31]. Our proteomics study carried out in collaboration with Harvard Proteomics Resource on the CSC-enriched versus differentiated PC3MM2 cells revealed 198 differentially expressed proteins out of 600 analyzed ones (unpublished data). Among them were many proteins involved in stem cell function, cell motility, invasion, metastasis and poor prognosis, including vimentin, nestin, S100, heat shock proteins HS90A and HS90B, moesin, galectin and many others. To determine possible drug-induced alterations in stemness gene expression, we analyzed CD133^+^ PPT2 cells before and after treatment with the SBT-1214/CMC 2.24 combination. Using PCR array assay (PAHS 501; SA*Biosciences*) with filtering criteria of a 1.5- or greater- fold change in expression, we have found that about 50% of the analyzed 84 stem cell-related transcription factors (TFs) were upregulated in CD133^+^ versus differentiated PrC cells (Figure 6A). Among them were CDX2, DLX2, DNMT3B, EGR, FOXP3, GLI2, HOX family TFs, IRX4, JUN, KLF2, NFATC1, NR2F2, PCNA, PITX3, POU4F1, SIX2, SOX2, SOX9, TERT, WT1 and others. Single treatment with SB-1214 (1µM) and CMC2.24 (10µM) for 24 hours induced significant down-regulation of these over-expressed genes (Figure 6B). Western blot analysis has shown that SBT-1214 and CMC2.24 as single agents induced moderate down-regulation of c-Myc and Sox2 in nuclear extracts of both CD133^+^ and bulk PPT2 cells, whereas combined drug administration led to practically complete inhibition of their expression (Figure 6C). Importantly, nuclear fractions of both CD133^+^ and bulk PPT2 cells did not express the two tumor suppressors/regulators of apoptosis, p53 and p21 (Figure 7A), which partially can explain their extreme resistance to anti-cancer drugs. Both SBT-1214 and CMC2.24, and especially their combination induced expression of p21 and p53. Such “gene wake-up” induced by pretreatment with the drug combination (SB-1214; 1µM and CMC2.24; 10µM) for 24 hours dramatically increased further sensitivity of these highly drug-resistant cells to the second treatment, leading to virtually complete death of the CSC-enriched cells (Figure 7B, C).

## Discussion

Accumulated data suggest that the tumor-initiating cells, CSCs, are not only highly resistant to conventional therapeutic strategies, but may actually promote cancer progression due to the drug-induced compensatory increase in their self-renewal [6-13]. Therefore, the search for effective therapeutic interventions should be based on the evaluation of the post-treatment status of the tumor-initiating CSCs, and not only on tumor shrinkage. In our studies, a high combined expression of CD133 and CD44 was selected as the first criterion for the isolation and initial enrichment of prostate CSCs. However, it is important to mention that further stringent stem cell-promoting culturing conditions are necessary for the maintenance and propagation of prostate CSCs. Such conditions include growing on type I collagen-coated surface or in 3D type I collagen-containing culture, in stem cell serum-free medium, at low cell density and with repeated sets of cell sorting. The stemness features of cells grown under these conditions were demonstrated in our previous functional and genomics studies [25,30-32]. In particular, we have found the overactivation of several developmental cascades, such as Hedgehog, EGFR, Wnt/β-catenin and Notch, which was linked with prostate stem cell regulation [33-35] and the progression of prostate cancer to androgen-independence and metastasis [36,37].

Currently, consensus does not exist regarding the phenotype of functionally distinctive subpopulations of normal versus cancerous cells in general, and normal stem cells versus CSCs in particular. However, accumulated data suggest that tumor-initiating cells can represent a heterogeneous population. It was shown recently that Epcam ^+^ /CD44^+^ and Epcam ^+^ /CD44^+^/CD49f^high^ basal cells can form abundant PrC spheres; in contrast, Epcam ^+^ /CD44^−^ cells cannot form spheres, but possess the Epcam ^+^ /CD44^−^/CD49f^high^ subpopulation with a basal profile similar to Epcam ^+^ /CD44^+^/CD49f^high^ sphere-forming cells, which are p63 ^+^ /AR ^low^/PSA^−^ [23]. After >10 passages, the majority of the PPT2 cells expressed cell surface markers commonly used for the isolation of tumor-initiating cells from different types of human cancer, including CD133, CD44, CD44v6, EpCAM, CD166, CD49f, and presently, they retain these features after more than 26 passages. The CD133^+^ PPT2 cells possess up-regulated levels of the majority of stem cell-related genes and several key transcription factors that maintain embryonic stem cells in a pluripotent state, including Sox-2, Oct-4 and c-Myc. The majority of these cells express high levels of nestin and vimentin, the cytoskeletal intermediate filament proteins characteristic for neural stem/progenitor cells, which also suggests their immature stem-like state. The involvement of SOX2 and OCT3/4 in prostate metastasis was demonstrated by targeted knockdown of these genes, which markedly suppressed the invasion of prostate cancer cells in vitro [38]. Accumulating data also suggest that both vimentin and nestin may be the key players in the transition from androgen-dependent to castration-resistant metastatic prostate cancer [39,40]. Importantly, the PPT2 is the first reported spontaneously immortalized prostate cancer cell line established from a primary prostate tumor, which possesses profound stemness characteristics, including up-regulated stemness genes, high sphere-forming, clonogenic and tumorigenic capacities and high resistance to chemotherapeutic drugs. In contrast to the previously reported spontaneously immortalized prostate cancer cell line Bob [41], which expresses high levels of p53 and markers of early differentiation—including K8, prostatic acid phosphatase and prostate stem cell antigen—the PPT2 line is negative for p53 and p21. In contrast to the original tumor tissue, the CD133^+^ PPT2 cells did not express AR and PSA, which is in line with other reports [21,42,43], and only a small fraction of the PPT2 cells express p63. A previous report has shown that all CD133^+^ cells isolated either from human telomerase reverse transcriptase (hTERT)-immortalized primary nonmalignant, or patient-derived malignant prostate epithelial cell lines retain stem cell properties with a CD133^+^/CD44^+^/α_2_β_1_^+^ /34βE12 ^+^ / /CK18 ^+^ /p63^-^/AR^-^/PSA^−^ phenotype [19]. It is widely accepted that, in contrast to normal prostate, malignant tumors do not contain basal cells, or at least do not express their common markers [44-47]. The functions of standard and variant CD44 isoforms remain unclear; however, their altered expression was demonstrated in several malignancies, including PrC [48]. In particular, CD44v6 was previously suggested as an independent indicator of poor prognosis in several cancer types and as a biological marker for the degree of malignancy [49]. In normal prostate glands, only rare basal and intermediate cells showed CD44 and CD44v6 labeling [50].

The morphological manifestation of CSCs is not well studied, partly because of the rarity of these cells and a lack of highly specific markers. However, accumulating data demonstrate that gigantic multinucleated cells, which are present in relatively low numbers in malignant tissues and cell lines, and can be enriched after treatment with standard anti-cancer drugs [31,51], possibly represent a subpopulation of cancer stem-like cells. Therefore, thorough analysis of such survivor cells is necessary for the identification of signaling pathways playing important roles in their biology and drug resistance. We observed especially strong expression of nestin and vimentin in such gigantic multinuclear treatment-survivor cells, which suggests that the most drug-resistant cells are at the immature state. Our data are in line with a recent report suggesting nestin as a novel target for metastatic pancreatic cancer [52]. Higher expression of nestin and CD133 on circulating melanoma cells was suggested as an index of poor prognosis [53]. All of the above suggests that the PPT2 cells represent a unique pre-clinical model for CSC-targeted drug development and basic functional studies of prostate cancer development.

Microtubule stabilizers such as paclitaxel (Taxol) and docetaxel can be initially effective in treating patients with androgen-independent prostate cancer, but the cancer almost invariably recurs in a more aggressive form [1,3,4]. Previously we have shown that the new-generation taxoid SBT-1214 induced complete regression of drug-resistant colon tumor xenografts in all surviving mice with unusually long-term tumor growth delay (>167 days) [24]. Later, we have found that this drug molecule induced significant down-regulation of multiple stemness-related genes, including several key transcription factors involved in the regulation of stem cells, cancer development and progression [25]. Of note, it was shown that docetaxel, which is the standard first-line therapy in metastatic castration-resistant prostate cancer, can promote drug resistance and de-differentiation (epithelial to mesenchymal transition; EMT) of the DU-145 and PC-3 cells via TGF-beta mechanism [54]. Cytotoxicity against prostate cancer cells can be significantly reduced with a combination of Paclitaxel and cyclopamine, a natural steroidal alkaloid that inhibits the Hedgehog pathway [55]. The anti-cancer effects of another natural phytochemical, curcumin (diferuloylmethane) were recently demonstrated in a large number of studies. Of great therapeutic interest, curcumin has been reported to induce the anti-proliferative and apoptotic effects on drug-resistant stem-like cells, and to improve the cytotoxic effects induced by diverse chemotherapeutic drugs [56-59]. However, the poor systemic bioavailability of curcumin and its high metabolic instability limit its therapeutic efficacy. Therefore, it was of great clinical significance to test the efficacy of CMC2.24, which has no systemic toxicity and has much higher bioavailability and activity compared to curcumin [29]. This prompted us to study the effect of combining SBT-1214 with CMC2.24. We have determined that a combination of the relatively low concentrations of SBT-1214 (0.1-1 µM) with CMC2.24 (30-40 µM) induced up to 80-90% death of the highly tumorigenic and highly drug-resistant prostate CD133^+^ cells maintained under stemness-promoting culture conditions. In addition, the significant up-regulation of the previously absent expression of the pro-apoptotic proteins, p53 and p21 (“gene wake-up” effect), and as a result, a dramatic increase in sensitivity to the following treatment leads us to believe that this drug combination can potentially reverse sensitivity to other anti-cancer drugs. Similar effects were reported for curcumin, which has been shown to activate p21 in breast [60], prostate [61] and colon [62] cancer cell lines. In earlier studies, the reduced expression of p21 in prostate cancer was associated with poor survival outcome [63,64]. The fact that the SBT-1214/CMC2.24 combination exerted high direct cytotoxicity and down-

regulated the stemness state of highly tumorigenic and clonogenic patient-derived CD133^+^ PPT2 cells is highly promising, because it potentially can also be effective against other patient-derived prostate CSC populations. Of interest, our genomic analysis (which is out of the scope of the present study) revealed that in addition to the lack of p53 and p21 expression, purified CD133^+^ PPT2 cells possess deletion of the Y chromosome, which was previously shown to be associated with the simultaneous inactivation of tumor suppressor genes [65,66]. Numerical abnormalities detected in the X chromosome in our CSC-enriched populations are also in line with previous reports, which have shown that such changes are present in 40% of prostate cancer cases [67,68] and are associated with hormone-refractory prostate cancer [69,70]. In general, loss of the Y chromosome and numerical abnormalities of the X chromosome are frequent phenomena observed in male patients with different cancer types [71], and especially with prostate cancer [65-70].

In conclusion, our data indicate that the anti-cancer efficacy of SBT-1214 *in vitro* and *in vivo* is most likely due to the down-regulation of multiple stem cell-related genes in the tumorigenic cell population (in addition to its known efficacy as a mitotic poison against proliferating cancer cells). We also demonstrate here that a combination of SBT-1214 with CMC2.24 exerts more profound pleiotropic, pan-inhibitory effects on a large number of stemness genes and transcription factors. In particular, modulation of multiple stem cell-relevant transcription factors and the pro-apoptotic p21 and p53 “gene wake-up” mechanism can potentially reverse resistance of CSCs to anti-cancer treatment and improve clinical outcome. We believe that evaluation of the two proprietary drugs, which exert pleiotropic CSC-targeted activities against primary patient-derived, spontaneously immortalized, low passage, highly tumorigenic and clonogenic prostate cancer cells with CD133^+/high^/CD44 +/high/CD49f+^/ high^/EpCAM^+/ high^ phenotype is clinically relevant and has high potential as a novel anti-cancer drug combination.

## Methods

### Prostate Tissues, Cells and Cultures

Human prostate needle biopsies were taken from otherwise discarded surgical waste in accordance with the SBU IRB and NIH requirements, via a research protocol that was approved by Stony Brook University Committees on Research Involving Human Subjects (CORIHS). Informed written consent was obtained on all participants. The PPT2 prostate adenocarcinoma cell line was established in March 2011 from the stage pT2c pNX pMX prostate cancer patient as a part of routine care for prostate cancer. Needle biopsies were mechanically and enzymatically disaggregated into single cell suspension at sterile conditions, rinsed with Hank’s balanced salt solution and incubated for 1.5 hours at 37°C in serum-free RPMI medium 1640 containing 200 units/ml Collagenases type II and type IV (Sigma-Aldrich), 120 µg/ml penicillin and 100 µg/ml streptomycin. Cells were further disaggregated by pipetting and serial filtration through cell dissociation sieves (size 40 and 80 meshes; Sigma-Aldrich). Single cell suspensions were placed on type-I collagen-coated dishes (Biocoat; Becton Dickenson, Bedford, MA) in serum-free Mesenchymal Stem Cell Growth Medium (MSCGM; Lonza) or Stemline Pluripotent Culture Medium (SPCM; Sigma-Aldrich). Fast adherent cells (within the following 15-20 min) were either collected and placed on ultra-low-adherent (ULA) plates or flasks (Corning) to induce floating 3D spheroids, or remained on the type I collagen-coated dishes for further propagation. Penicillin, streptomycin and TrypLE were obtained from Invitrogen (Grand Island, NY, USA). The MSCGM and SPCM were changed twice weekly. All experiments were performed on primary prostate cancer patient-derived PPT2 cells and for comparison, on the established highly metastatic derivative of the androgen-independent PC-3 cell line, PC3MM2, originally purchased from the M. D. Andersen Cancer Center, TX.

### Isolation, Purification and Characterization of the Tumor-Initiating Cells

To ensure more reliable isolation of CSCs, cells were labeled with one or several markers conjugated with different fluorescent dyes, including anti-human CD133/2-APC (clone 293C3; Miltenyi Biotec, CA, USA); CD166-PE (clone 105902; R&D Systems, MN, USA); CD44-FITC (clone F10-44-2), CD44-PE (clone F10-44-2; Invitrogen/Biosources, USA); CD44v6-FITC (clone 2F10; R&D Systems, USA), EpCAM-FITC (Biosource, CA, USA), Pan-Keratin (C11) -Alexa Fluor^®^ 488 (Cell Signaling) and all the isotype controls (Chemicon). Antibodies were diluted in buffer containing 5% BSA, 1mM EDTA and 15-20% blocking reagent (Miltenyi Biotec) to inhibit unspecific binding to non-target cells. After 15 min incubation at 4°C, stained cells were sorted and analyzed with multiparametric flow cytometer BD FACS*Aria* (Becton Dickinson, CA). Alternatively, dissociated cells were centrifuged at 950 g for 5 min at 4°C, rinsed with sterile MACS buffer (Miltenyi Biotec, CA) and labeled with CD133 Abs directly or indirectly conjugated with ferromagnetic beads (Miltenyi Biotec, CA) as recommended by manufacturer.

### Sphere Formation Assay

In order to obtain prostate cancer spheroids, MACS-CD133 and MACS-CD44 (Miltenyi Biotec, CA) or FACS sorted CD133^high^/CD44^high^ cells were seeded at low numbers on ULA plates (1,000 cells per 6-well plate or 5-10,000 cells per 75 mm flasks) in MSCGM or SPCM. For evaluation of the clonogenic/sphere-forming capacity, cells were counted with Cellometer Auto T4 (Nexcelom Bioscience LLC, MA), resuspended in 1:4 type I collagen/SPCM or 1:4 Matrigel/MSGM and known cell numbers were plated on ULA plates. One week after initiation plates were inspected for floating sphere growth and compact well-shaped spheroids were counted. Spheroids were serially passaged by gentle dissociation and mixing with a new Matrigel/MSGM or type I collagen/SPCM, or reseeded on 48-well plates for final count of 5-10 spheres per well for further propagation and analysis.

### Mice Tumor Xenografts

All experiments involving the use of animals were carried out in strict accordance with the recommendations in the Guide for the Care and Use of Laboratory Animals of the National Institutes of Health, via a research protocol that was approved by Stony Brook University Institutional Animal Care and Use Committee (IACUC). NOD/SCID mice (Charles River Laboratories International, Inc., MA) were maintained under defined conditions at SBU animal facility. After sufficient propagation, CD133^+^ cells were resuspended in 1:1 MSCGM/Matrigel and injected to the flanks of 6 weeks old NOD/SCID mice (3,000 cells per mice; subcutaneously). Tumor development was monitored starting from the second week. The primary tumor sizes were measured with a caliper on a weekly basis and approximate tumor weights determined using the formula 0.5ab^2^, where b is the smaller of the two perpendicular diameters. All mice were terminated after fourth week of treatment, if the tumor measured ~2 cm, or at the first sign of suffering.

Ability to induce the round colonies (holoclones) was also determined before and after drug treatment. Cells were counted and plated on 48-well plates at a final count of 300 cells per well. One week after initiation, the plates were inspected for colony growth and the number of colonies within each well was quantified by phase contrast microscopy. After drug treatment, one portion of cells which survived particular treatment regimen was placed at 3D and adherent culture conditions and analyzed. Another portion was used for analysis of the drug-induced alterations in expression of the stem cell markers with PCR array assay, western blotting and FACS.

Ability to induce the round colonies (holoclones) was also determined before and after drug treatment. Cells were counted and plated on 48-well plates at a final count of 300 cells per well. One week after initiation, the plates were inspected for colony growth and the number of colonies within each well was quantified by phase contrast microscopy. After drug treatment, one portion of cells which survived particular treatment regimen was placed at 3D and adherent culture conditions and analyzed. Another portion was used for analysis of the drug-induced alterations in expression of the stem cell markers with PCR array assay, western blotting and FACS.

### Drugs

Paclitaxel (Ptx; Taxol) was provided by Indena (Milan, Italy). New-generation taxoid, SB-T-1214 was synthesized by Dr. Ojima’s group (Stony Brook University, ICB&DD, NY, USA). Chemically modified curcumin, CMC-2.24 was synthesized by Dr. Johnson’s group (ChemMaster International, Inc., Stony Brook, NY, USA). DMSO and MTT were purchased from Fisher Scientific (Pittsburgh, PA, USA).

### *In vivo* Cytotoxicity of SBT-1214

Animals were handled according to protocols that have been approved by the Institutional Animal Care and Use committee of the Stony Brook University. The staff of veterinarians provided veterinary care. The SBT-1214 was administered intravenously (40, 20, 20, 20 mg/kg weekly) to 6-weeks old NOD/SCID mice. Treatment was started 2-3 weeks after transplantation of the tumor cells, when tumor xenografts reached ~100 mm^3^. Systemic toxicity in NOD/SCID mice was closely monitored and evaluated by standard criteria (motor activity, morbidity, appetite, posture and appearance). One week after the last treatment, all residual tumors were harvested and analyzed histopathologically and for *ex vivo* clonogenic capacity. One part of each mouse tumor xenograft was snap-frozen in embedding matrix (Lipshaw) and kept at -80°C before immunohistochemical analysis at standard conditions. Another portion of each harvested tumor was mechanically and enzymatically disaggregated into single cell suspension as previously described [18]. Ability to induce the round colonies (holoclones) was also determined before and after drug treatment. Cells were counted and plated on 48-well plates at a final count of 300 cells per well. One week after initiation, the plates were inspected for colony growth and the number of colonies within each well was quantified by phase contrast microscopy. After drug treatment, one portion of cells which survived particular treatment regimen was placed at 3D and adherent culture conditions and analyzed. Another portion was used for analysis of the drug-induced alterations in expression of the stem cell markers with PCR array assay, western blotting and FACS.

### *In vitro* Cytotoxicity

The CSC-specific cytotoxic effects were studied in two different settings both of which are known to maintain/promote stemness phenotype: adherent to type I collagen cultures and 3D cultures of floating spheroids [67-69]. The CD133^high^/CD44^high^ cells (1 × 10^3^ cells/well) isolated either from PPT2 or PC3MM2 cell lines were seeded onto the type I collagen-coated 96-well plates, cultured in the MSCGM for 2 days, and the treatment was initiated upon 90% confluency. Paclitaxel, SBT-1214 and CMC2.24 were dissolved in sterile DMSO and then serially diluted in MSCGM. For testing the SBT-1214/CMC2.24 combination efficacy, the regular MSCGM was replaced with treatment medium containing SBT-1214 or Ptx at selected concentration range (from 10 nM to 10 µM) with 30 µM CMC2.24. Treatment medium was removed 24, 48 or 72 hr later, followed by return to the MSCGM. Cell death was analyzed on adherent type I collagen cell cultures with the MTT assay [MTT = 3-(4,5-dimethylthiazol-2-yl)-2,5-diphenyltetrazolium bromide] as recommended by manufacturer (Invitrogen). Spheroids were cultured and treated on ULA plates.

### PCR Array Assay

Stem cell-specific transcription factors gene expression profiles were studied with the PCR Array assay (SABiosciences, CA; PAHS-501) in accordance with the manufacturer’s recommendations. Briefly, total RNA was isolated from different cell populations or whole floating spheroids using PARIS kit (Ambion). Up to 1 µg of total RNA was treated with DNase and cDNA was prepared using RT^2^ First Strand kit. For each analysis, pairs of the test and control cDNA samples were mixed with RT^2^ qPCR Master mix and distributed across the PCR array 96-well plates, each of which contained 84 stem cell-related probes and control housekeeping genes. After cycling with real-time PCR, obtained amplification data (fold-changes in C_t_ values of all the genes) was analyzed with the SABiosciences software.

### Western Blot Analysis

Cells were lysed using Active Motif Nuclear Co-ip kit according to manufacturer’s protocol. 20µg of enriched nuclear and cytoplasmic extracts was subjected to electrophoresis in 10% polyacrylamide gel. The proteins were then transferred to a nitrocellulose membrane and developed with appropriate antibodies. Mouse monoclonal antibody against Histone H1 (05-457) and GAPDH (MAB374) were purchased from Millipore and mouse monoclonal antibody against p21 was purchased from BD Pharmingen (556431). Rabbit antibody against p53 was purchased from Santa Cruz Biotechnology (sc-6243). Rabbit antibodies against Sox2 (2748), C-Myc (5605), and Oct4 (2750) were purchased from Cell Signaling.

### Statistical Analysis

The dose-response cytotoxicity of SBT-1214, CMC2.24/and their combination was evaluated by standard MTT assay (all of the *in vitro* experiments were repeated at least three times and each measurement for the MMT assay was performed in 4 repeats). The dose-response points were plotted as a percentage of the untreated control, the absorbance of which was considered as 100%. Data were expressed as means ± SD for each drug concentration. The *in vivo* responses to drug treatment were evaluated as changes in tumor volume of drug-treated versus untreated control xenografts induced by particular type of cells. Data were expressed as means ± SD for control and drug treated tumors. The statistical significance of differences was determined using Student’s *t*-test. The parameters used were the two-tailed distribution and the paired test.

*P*<0.05 was considered statistically significant.
